# Antioxidant and Antidiabetic Activity of Proanthocyanidins from *Fagopyrum dibotrys*

**DOI:** 10.3390/molecules26092417

**Published:** 2021-04-21

**Authors:** Xin Li, Jingling Liu, Qinxiang Chang, Ziyun Zhou, Ruilian Han, Zongsuo Liang

**Affiliations:** 1 College of Life Sciences, Northwest A & F University, Yangling 712100, China; lixin0715@nwafu.edu.cn (X.L.); jinglingliu-sm@nwsuaf.edu.cn (J.L.); xnzzy@nwsuaf.edu.cn (Z.Z.); 2 Institute of Landscape, Taiyuan University, Taiyuan 030032, China; changqxkys@tyu.edu.cn; 3 Zhejiang Provincial Key Laboratory of Plant Secondary Metabolism Regulation, College of Life Science and Medicine, Zhejiang Sci-Tech University, Hangzhou 310018, China; hanrl@nwsuaf.edu.cn

**Keywords:** *Fagopyrum dibotrys*, proanthocyanidins, antioxidant, antidiabetes, structural analysis

## Abstract

Proanthocyanidins are natural glycosidase inhibitors with excellent antioxidant activity. This study aims to search for a new source of proanthocyanidins for the prevention and treatment of type 2 diabetes with higher content and better activity and get their structure elucidated. First, the total proanthocyanidins contents (TOPCs), antioxidant activity, antidiabetic activity of seven common Polygonaceae plants were analyzed and compared. Then proanthocyanidins from the rhizome of *Fagopyrum dibotrys* were purified, and the detailed structure was comprehensively analyzed by ultraviolet visible spectroscopy (UV-Vis), Fourier transform infrared spectroscopy (FT-IR), ^13^C nuclear magnetic resonance spectroscopy (^13^C NMR), reversed-phase high-performance liquid chromatography-electrospray mass spectrometry (RP-HPLC-ESI-MS), and matrix-assisted laser desorption/ionization-time of flight mass spectrometry (MALDI-TOF MS). The rhizome of *F. dibotrys* showed the highest TOPCs, the strongest antioxidant, and antidiabetic activities; the TOPCs, antioxidant and antidiabetic activities were all very significantly positively correlated. Proanthocyanidins purified from the rhizome of *F. dibotrys* showed better antidiabetic activity than grape seed proanthocyanidins (GsPs). Seventy-two proanthocyanidins from trimer to undecamer with a mean degree of polymerization (mDP) of about 5.02 ± 0.21 were identified with catechin and epicatechin as the dominant monomers. Conclusion: Proanthocyanidins are the main antioxidant and antidiabetic active substances of *F. dibotrys* and are expected to be developed into potential antioxidant and hypoglycemic products.

## 1. Introduction

In recent years, the incidence of type 2 diabetes has been increasing year by year and remains high in the world, which brings great medical and economic burden to society [1]. Glycosidase inhibitors can effectively inhibit the digestion and absorption of sugars in the digestive tract and are highly sought after by diabetic patients because of their high safety and low side effects [2,3,4,5]. In addition, antioxidant supplementation can effectively reduce the risk and improve the symptoms of type 2 diabetes and its complications [1,4,5,6]. Hence, the development of natural glycosidase inhibitors with excellent antioxidant activity has become a hot topic of scientists in recent years [2,3,4,5].

Proanthocyanidins are plant polyphenols formed by the condensation of flavane-3-ol monomers such as catechin, epicatechin as shown in Figure S1; the diversity of monomer type, composition, substituent group on monomer, linkage-type, and the degree of polymerization together made proanthocyanidins diverse in structure and activity [6]. Proanthocyanidins are widely used in medicine, functional foods, and daily chemical product additives due to their biological activities such as antioxidant, antidiabetes, anti-inflammatory, antiallergic, and antimicrobial, etc. [6,7,8]. Many of the biological activities of proanthocyanidins are mainly realized through their excellent antioxidant activity and interaction with proteins [9]. Proanthocyanidins have both excellent antioxidant activity and glycosidase inhibitory activity, which has been proved to be able to effectively relieve oxidative stress while preventing and treating diabetes [2,3,4,5]. Therefore, it is of great significance to search for natural plant resources of proanthocyanidins with high content, wide sources, and low cost and to elucidate their structural characteristics for the development of drugs for the prevention and treatment of type 2 diabetes [1,2,3,4,5,7].

The contents and bioactivities of proanthocyanidins in different parts of seven Polygonaceae plants, including *Fagopyrum dibotrys* [10], *Reynoutria japonica* [11], and *Rheum officinale* [12], were compared, and the rhizome of *F. dibotrys* was selected as the material for further study because it had the highest total proanthocyanidins content (TOPCs), the strongest antioxidant and antidiabetic activity among the seven Polygonaceae plants. *F. dibotrys*, also known as golden buckwheat, is a perennial erect herb of genus *Fagopyrum* in Polygonaceae, which mainly grows in the north temperate zone and is widely distributed in China, Kazakhstan, Russia, Ukraine, and other regions [10]. *F. dibotrys* has rich nutritional value and health care function, and the rhizome of *F. dibotrys* has a long history of being used as an anticancer and anti-inflammatory herb in China [10,13,14]. Since substances used in traditional medicine systems have long played a vital role in meeting global healthcare needs, so it is promising to find useful substances from *F. dibotrys* due to the longtime usage as folk medicine [15].

Modern studies have shown that the rhizome of *F. dibotrys* contains flavonoids, phenols, fagopyritols, triterpenoids, fatty acids, and steroids [10,16,17,18,19], which have bioactivities such as antitumor [20,21,22], anti-oxidation [13,14,23], antidiabetes [24,25,26,27] and so on. At present, there are few reports on proanthocyanidins of *F. dibotrys* [10]. It has been reported that proanthocyanidins were isolated from the rhizome of *F. dibotrys* [10,28]. However, the bioactivity, structural composition, polymerization degree distribution of polymeric proanthocyanidins, and other information of proanthocyanidins of *F. dibotrys* have not been reported yet.

In this study, proanthocyanidins of *F. dibotrys* were used as the research object to analyze the correlation between proanthocyanidins and antioxidant and antidiabetic activities. In addition, ultraviolet visible spectroscopy (UV-Vis) [4,29], Fourier transform infrared spectroscopy (FT-IR) [4,29], ^13^C nuclear magnetic resonance spectroscopy (^13^C NMR) [30,31], reversed-phase high-performance liquid chromatography-electrospray mass spectrometry (RP-HPLC-ESI-MS) [31,32,33] and matrix-assisted laser desorption/ionization-time of flight mass spectrometry (MALDI-TOF MS) [31,32,33] were comprehensively used to analyze the structure of proanthocyanidins purified from *F. dibotrys*. It is hoped that this study can provide a reference for the development of the antioxidant and antidiabetes products of *F. dibotrys*.

## 2. Results and Discussion

### 2.1. Total Proanthocyanidins Content (TOPCs), Antioxidant and Antidiabetic Activities of Seven Polygonaceae Plants 

The TOPCs, antioxidant and antidiabetic activities of different parts of the seven Polygonaceae plants were analyzed and compared with grape seed as control. The TOPCs of the seven Polygonaceae plants and grape seeds were shown in Table 1. The results showed that proanthocyanidins were detected in all the samples, but The TOPCs in different samples were significantly different. The rhizome of *R. officinale*, the rhizome of *R. japonica*, the rhizome, and shoots of *F. dibotrys* all contained more than 10 mg GsPs /g DW of TOPCs, and the rhizome of *F. dibotrys* was the highest, which was 3.70 and 6.21 times of the rhizome of *R. officinale* and *R. japonica*, and 1.09 times of grape seed, respectively. In addition, the TOPCs of the rhizome of *F. dibotrys* were about 6.49 times that of the shoots of *F. dibotrys*. The results showed that the content of proanthocyanidins not only varied in different plants of the same family but also varied in different parts of the same plant [13,34].

The antioxidant activities of the seven Polygonaceae plants and grape seeds are shown in Table 2. The DPPH free radical scavenging rate and CUPRAC value of the rhizome of *F. dibotrys* were significantly higher than those of grape seed and other Polygonaceae plants. The ABTS free radical scavenging rate and FRAP value of the rhizome of *F. dibotrys* were about 1.01 and 0.89 times those of grape seed and significantly higher than those of other Polygonaceae plants. The order of antioxidant activity of different samples was relatively consistent with their TOPCs.

The antidiabetic activities of the seven Polygonaceae plants and grape seeds are shown in Table 3. The inhibitory activities of the rhizome of *F. dibotrys* against *S. cerevisiae α*-glucosidase, porcine pancreatic *α*-amylase, and human salivary *α*-amylase were significantly stronger than those of other Polygonaceae plants. Moreover, the inhibitory activities of the rhizome of *F. dibotrys* against *S. cerevisiae α*-glucosidase, porcine pancreatic *α*-amylase, and human salivary *α*-amylase were 0.86, 0.97, and 1.15 times of those of grape seed, respectively, indicating that the rhizome of *F. dibotrys* and grape seed had similar antidiabetes activity. Similar to the results of antioxidant analysis, the order of antidiabetic activities of different samples was also in high consistency with their respective TOPCs.

As shown in the above tables, the rhizome of *F. dibotrys* showed the highest TOPCs, the strongest antioxidant activity and the strongest antidiabetic activity among the seven Polygonaceae plants. Moreover, the TOPCs, antioxidant activity and antidiabetes activity of the rhizome of *F. dibotrys* were similar to those of grape seed. Vast studies showed that the main components of the antioxidant and antidiabetic activities of grape seed were proanthocyanidins [1,2,3,4,5,6,7,8,9,10,34,35]. In addition, this study found that the order of TOPCs of the samples was highly consistent with the order of all the antioxidant and antidiabetic indexes. To better explore the correlation between TOPCs and antioxidant and antidiabetic activities in these Polygonaceae plants, Pearson correlation coefficient between TOPCs and antioxidant and antidiabetic activity in each sample was analyzed and the results were shown in Table 4. The results showed that TOPCs were significantly positively correlated with antioxidant and antidiabetic activities, and all the antioxidant activities and antidiabetic activities were significantly positively correlated with each other. All the correlation coefficients reached the *p* < 0.01 level, indicating that proanthocyanidins may be important antioxidant and antidiabetic compounds in these Polygonaceae plants.

### 2.2. Purification and Bioactivity of Proanthocyanidins from the Rhizome of F. dibotrys

The above studies showed that the rhizome of *F. dibotrys* contained the most abundant proanthocyanidins and exhibited the strongest antioxidant and antidiabetic activities among the selected Polygonaceae plant samples. To better reveal the relationship between the TOPCs and its biological activity in the rhizome of *F. dibotrys*, a 50% methanol eluent and a 70% acetone eluent were successively obtained from the crude extract of the rhizome of *F. dibotrys* by Sephadex LH-20 and the TOPCs of the three extracts were determined and were shown in Table 5. The TOPCs of the 50% methanol eluent, crude extract and the 70% acetone eluent of the crude extract of the rhizome of *F. dibotrys* increased in turn, and the TOPCs of the 70% acetone eluent was 4.99 times and 79.48 times of crude extract and the 50% methanol eluent respectively, indicating that Sephadex LH-20 could effectively achieve the enrichment of proanthocyanidins in crude extract of the rhizome of *F. dibotrys*.

The antioxidant activities of the three extracts were shown in Table 6. The DPPH radical scavenging activity, ABTS radical scavenging activity, FRAP antioxidant activity and CUPRAC antioxidant activity of the 50% methanol eluent, crude extract and the 70% acetone eluent of the rhizome of *F. dibotrys* were all increased successively, which were consistent with the order of their TOPCs. The DPPH free radical scavenging activity, ABTS free radical scavenging activity and CUPRAC antioxidant activity of the 70% acetone eluent were significantly higher than those of Vc and Trolox, and the antioxidant activities of the 70% acetone eluent were 2.92~3.36 times those of crude extract, 5.27~6.28 times those of the 50% methanol eluent, and 0.93~1.05 times those of GsPs, indicating that the antioxidant activities of proanthocyanidins obtained from the crude extract of the rhizome of *F. dibotrys* had been enriched by Sephadex LH-20 and were close to those of GsPs.

The antidiabetic activities of the 50% methanol eluent, crude extract and the 70% acetone eluent of the rhizome of *F. dibotrys* were shown in Table 7. The inhibitory activities of the 50% methanol eluent, crude extract and the 70% acetone eluent of the rhizome of *F. dibotrys* against *S. cerevisiae α*-glucosidase, porcine pancreatic *α*-amylase and human salivary *α*-amylase were all increased successively, which were consistent with the order of their TOPCs and antioxidant activities. The inhibitory activities of the 70% acetone eluent against the three glycosidases were 5.11~8.73 times of crude extract and 55.87~112.78 times of the 50% methanol eluent, and 1.09~1.18 times of GsPs. Moreover, the inhibitory activity of the 70% acetone eluent against *S. cerevisiae α*-glucosidase, was about 190.86 times that of acarbose. All these indicated that the proanthocyanidins obtained from the crude extract of the rhizome of *F. dibotrys* and enriched by Sephadex LH-20 had strong antidiabetic activities, and the activities were significantly higher than those of GsPs.

The comparison of TOPCs, antioxidant and antidiabetic activities of crude extract, the 50% methanol eluent and the 70% acetone eluent of the rhizome of *F. dibotrys* showed that all the antioxidant and antidiabetic indexes of the three extracts were enhanced with the increase in TOPCs, and the antioxidant activities of the 70% acetone eluent with the highest TOPCs were similar to those of GsPs, while the antidiabetic activities were significantly stronger than those of GsPs. All these further confirmed that proanthocyanidins were the main antioxidant and antidiabetic active substances in the rhizome of *F. dibotrys*.

### 2.3. Structure Analysis of Proanthocyanidins in the Rhizome of F. dibotrys

There have been no reports about the detailed structure of proanthocyanidins in the rhizome of *F. dibotrys* so far. To better study and develop the proanthocyanidins in *F. dibotrys*, it was important and necessary to analyze the structure of proanthocyanidins. Therefore, UV-Vis, FT-IR, ^13^C NMR, RP-HPLC-ESI-MS, and MALDI-TOF MS were used to analyze the structure of proanthocyanidins in the 70% acetone eluent of the rhizome of *F. dibotrys* in this study.

#### 2.3.1. UV-vis Analysis

As a class of compounds containing conjugated structures, the solution of proanthocyanidins has characteristic absorption peaks in the ultraviolet region. The ultraviolet spectrum of a typical proanthocyanidins solution should have two absorption peaks located near 230 nm and 280 nm, respectively. In general, the absorption peak near 280 nm is symmetric, and there should be no absorption peak in the wavelength range greater than 280 nm. Under the same mass concentration, the intensity of the absorption peak near 230 nm should be significantly greater than that near 280 nm [4,28]. The n-butanol-hydrochloric acid colorimetric method is the most classical and most widely recognized method for the analysis of proanthocyanidins. Proanthocyanidins can be hydrolyzed to produce anthocyanins in a hot acidic environment, while anthocyanins have an absorption peak near 550 nm in an acidic environment, so the purpose of analyzing proanthocyanidins can be achieved by analyzing the generated anthocyanins [34,35].

**Figure 1:**
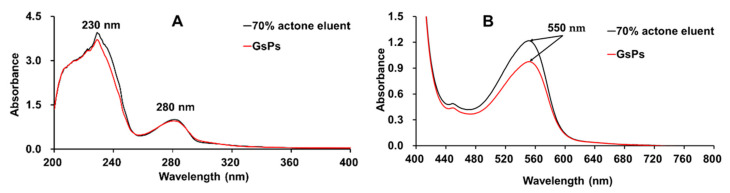
(**A**) The ultraviolet spectrum of the 70% acetone eluent of the rhizome of *F. dibotrys* and GsPs in methanol solution, (**B**) the visible spectrum of the 70% acetone eluent of the rhizome of *F. dibotrys* and GsPs after color development by n-butanol-hydrochloric acid colorimetric method.

In this study, GsPs were used as the control to compare the UV-Vis spectrum of the 70% acetone eluent of the rhizome of *F. dibotrys* under the same experimental conditions and with the same concentrations, and the results are shown in Figure 1. According to their respective spectral curves, both the 70% acetone eluent of the rhizome of *F. dibotrys* and GsPs exhibited the same characteristic absorption peaks, and no impurity peaks that should not appear were detected. The results showed that the 70% acetone eluent of the rhizome of *F. dibotrys* contained proanthocyanidins with relatively high purity, which was consistent with the previous results that the TOPCs of the 70% acetone eluent of the rhizome of *F. dibotrys* was as high as 1223.15 ± 20.64 mg GsPs /g DW [4,29,35,36].

#### 2.3.2. FT-IR Analysis

**Figure 2:**
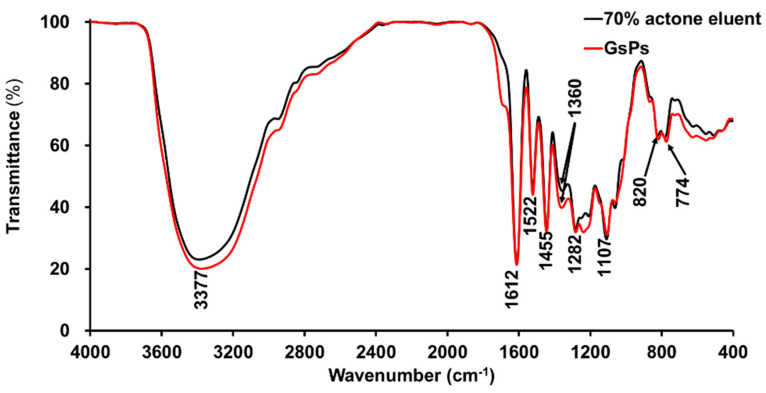
The FT-IR spectrum of the 70% acetone eluent of the rhizome of *F. dibotrys* and GsPs.

The FT-IR analysis can provide some functional group information of proanthocyanidins. The absorption peaks of hydroxyl and benzene ring skeleton on flavan3-ol monomers are the main characteristic absorption peaks of proanthocyanidins in the FT-IR spectrum [4,29]. In this study, the FT-IR spectrum of the 70% acetone eluent of the rhizome of *F. dibotrys* and GsPs were obtained, as shown in Figure 2.

In the spectrum, the absorption peak near 3377 cm^−1^ was the stretching vibration absorption peak of the hydroxyl group in phenolic molecules, and the less obvious absorption peak near 2900 cm^−1^ was the antisymmetric stretching vibration absorption peak of =C-H in the benzene ring. The less obvious absorption peak near 2850 cm^−1^ was the symmetric stretching vibration absorption peak of =C-H on the benzene ring. There was no significant absorption peak of the 70% acetone eluent of the rhizome of *F. dibotrys* near 1700 cm^−1^ in the spectrum, while GsPs could detect a relatively weak absorption peak, which was the carbonyl group signal on the galloyl group, indicating that the degree of gallic acylation of the 70% acetone eluent was relatively low compared with GsPs, so that it could not be detected by FT-IR. The absorption peaks near 1612,1522 and 1455 cm^−1^ were the skeleton stretching vibration absorption peaks of the benzene ring. The absorption peak near 1360 cm^−1^ came from the bending vibration absorption peak of phenolic hydroxyl group. The absorption peaks near 1282 and 1200 cm^−1^ were the symmetric and antisymmetric stretching vibration absorption peaks of C-O-C ether bond on the heterocycle. The absorption peak near 1107 cm^−1^ was the in-plane bending vibration absorption peak of =C-H on the benzene ring. The absorption peaks near 820 and 774 cm^−1^ were the out-of-plane bending vibration absorption peaks of =C-H on the benzene ring. In the fingerprint region of 1300–400 cm^−1^, the 70% acetone eluent and GsPs had similar absorption peaks but with some differences, which indicates that the 70% acetone eluent and GsPs should have a similar structure but with some differences and the detailed structure of the 70% acetone eluent of the rhizome of *F. dibotrys* needs to be further analyzed [4,29].

#### 2.3.3. ^13^C NMR Analysis

The ^13^C NMR of proanthocyanidins can provide abundant structural information of proanthocyanidins, including the composition, proportion, the three-dimensional configuration, and the linkage type of monomers, as well as the degree of polymerization of proanthocyanidins, etc. [9,30,31,32,33,37,38,39].

**Figure 3:**
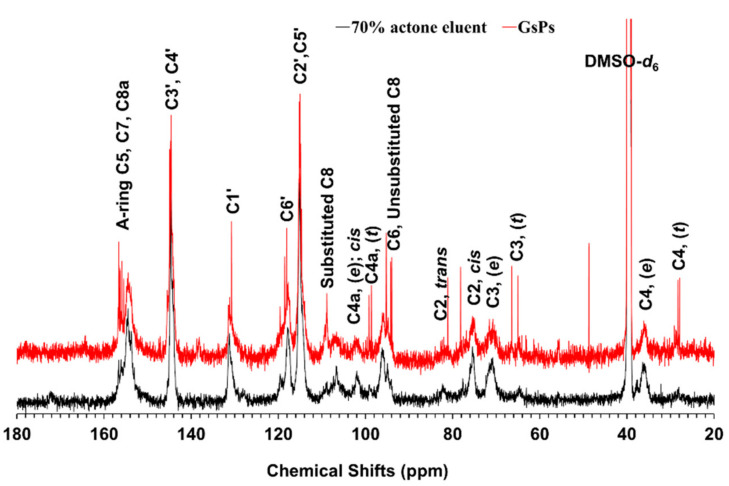
The ^13^C NMR spectrum the 70% acetone eluent of the rhizome of *F. dibotrys* and GsPs.

The ^13^C NMR spectrum analysis results of the 70% acetone eluent of the rhizome of *F. dibotrys* and GsPs are shown in Figure 3. The wide peaks between 157 and 150 ppm attributed to the signals of C5, C7, and C8a on the A ring, and the peaks between 145 and 115 ppm belonged to the signals of C3′, C4′, C1′, C6′, and the C2′ and C5′ signals of procyanidins on the B ring. The peaks between 110 and 90 ppm belonged to the signals of C8, C6, and C4a in the extension and terminal units. The peaks between 90 and 60 ppm belonged to the signals of C2 and C3 in the extension and terminal units. While the peaks near 36 and 28 ppm were the C4 signal of the extension units and terminal units. The peaks near 39.5 ppm were the carbon signals of the solvent DMSO-*d*_6_ [33,38,39].

By comprehensively analysis of the ^13^C NMR spectrum of the 70% acetone eluent of the rhizome of *F. dibotrys* and GsPs, it can be concluded that the proanthocyanidins in the 70% acetone eluent of the rhizome of *F. dibotrys* and GsPs were similar in structure and were mainly composed of (epi)catechin monomers, no obvious (epi)gallocatechin monomers, gallic acylated monomers, and A-type linkage were detected [9,38,39]. The 2′3-*cis* configuration was dominant, and the ratio of 2′3-*cis* to 2′3-*trans* configuration was about 4:1 [9]. According to the ratio of the peak areas of C3 and C4 in the extension and terminal units, the mean degree of polymerization (mDP) of proanthocyanidins in the 70% acetone eluent was estimated to be about 5 [9].

#### 2.3.4. RP-HPLC-ESI-MS Analysis of Thiolytic Degradation Products

Under acidic conditions, the nucleophile benzyl mercaptan can degrade the proanthocyanidins, the terminal units are released in the form of flavane-3-ols, while the extension units can form the corresponding benzyl thioether with benzyl mercaptan. During the degradation of proanthocyanidins by benzyl mercaptan, the three-dimensional configurations of C2 and C3 on the C-ring of the monomers were not affected, and the C-C and C-O-C double bonds in the A-type linkage were not affected and retained. Therefore, the analysis of thiolytic degradation products can deduce the structure of proanthocyanidins backward so as to obtain the specific composition of terminal units and extension units as well as the polymerization degree information, which is considered to be an effective method to analyze the structure of proanthocyanidins [30,31,32,33,34].

**Figure 4:**
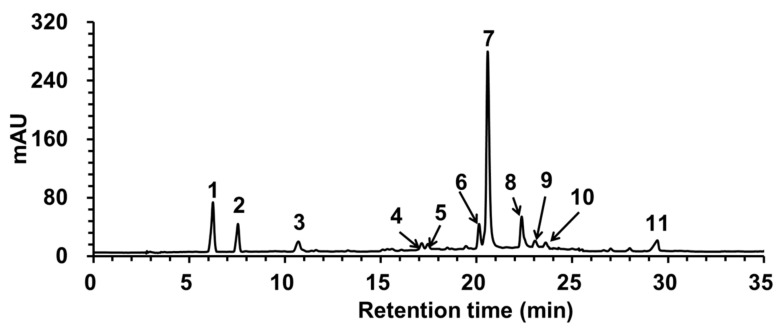
The RP-HPLC spectrum of thiolytic degradation products of the 70% acetone eluent of the rhizome of *F. dibotrys*.

In this study, RP-HPLC-ESI-MS was used to analyze the thiolytic degradation products of the 70% acetone eluent of the rhizome of *F. dibotrys*. The HPLC spectrum of thiolytic degradation products are shown in Figure 4. The molecular ions [M − H]^−^ of peaks 1-3 were *m*/*z* 289, 289, and 441, respectively; by comparing the retention time and mass spectrum data of standard references, it was determined that peaks 1–3 were catechin, epicatechin, and epicatechin gallate, respectively, which were the terminal units [33,34]. While the molecular ions [M − H]^−^ of peaks 4–11 were *m*/*z* 427, 427, 411, 411, 563, 697, 395, and 123, respectively, by comparing the mass spectrum data and combining with the reference literature [34], it was determined that peaks 4–11 were gallocatechin benzyl thioether, epigallocatechin benzyl thioether, catechin benzyl thioether, epicatechin benzyl thioether, (epi)catechin gallate benzyl thioether, the benzyl thioether of an A-type dimer composed of two (epi)catechin monomers, (epi)afzelechin benzyl thioether and excessive benzyl mercaptan, respectively. So, the extension units were epigallocatechin, gallocatechin, catechin, epicatechin, (epi)catechin gallate, (epi)afzelechin, and A-type dimer, respectively [33].

The structural composition of proanthocyanidins in the 70% acetone eluent of the rhizome of *F. dibotrys* is shown in Table 8. Catechin and epicatechin together constituted about 90% of the monomers, and the A-type linkages existed only in the extension unit, which accounted for 0.78% of all the monomers was also composed of two (epi)catechin monomers, but the proportion of catechin and epicatechin in the extension units and terminal units were significantly different. The 2′3-*cis* configuration was dominant, and the proportion of 2′3-*cis* configuration was about 79.13% ± 0.85%, which was consistent with the ^13^C NMR result that the ratio of 2′3-*cis* to 2′3-*trans* configuration was about 4:1. The calculated mDP was about 5.02 ± 0.21, which was also consistent with the ^13^C NMR analysis results. Owing to the higher sensitivity of RP-HPLC-ESI-MS, (epi)catechin gallate was detected from both extension and terminal units, indicating a certain degree of gallic acylation of monomers [33]. Meanwhile, gallocatechin and epigallocatechin were detected from the extension units, and the presence of (epi)afzelechin was also detected from the extension units. In conclusion, the above structural information should provide a reference for the subsequent MALDI-TOF MS analysis.

#### 2.3.5. MALDI-TOF MS Analysis

In this study, ^13^C NMR and RP-HPLC-ESI-MS were used to obtain the monomer types, the composition of terminal units and extension units, the mDP, the spatial configuration, and the substituents of the monomers of proanthocyanidins in the 70% acetone eluent of the rhizome of *F. dibotrys*. On this basis, MALDI-TOF MS was used to analyze the distribution of polymeric proanthocyanidins and the linkage types of these polymeric proanthocyanidins, and more comprehensive and detailed structural information was obtained [31,32,33].

**Figure 5:**
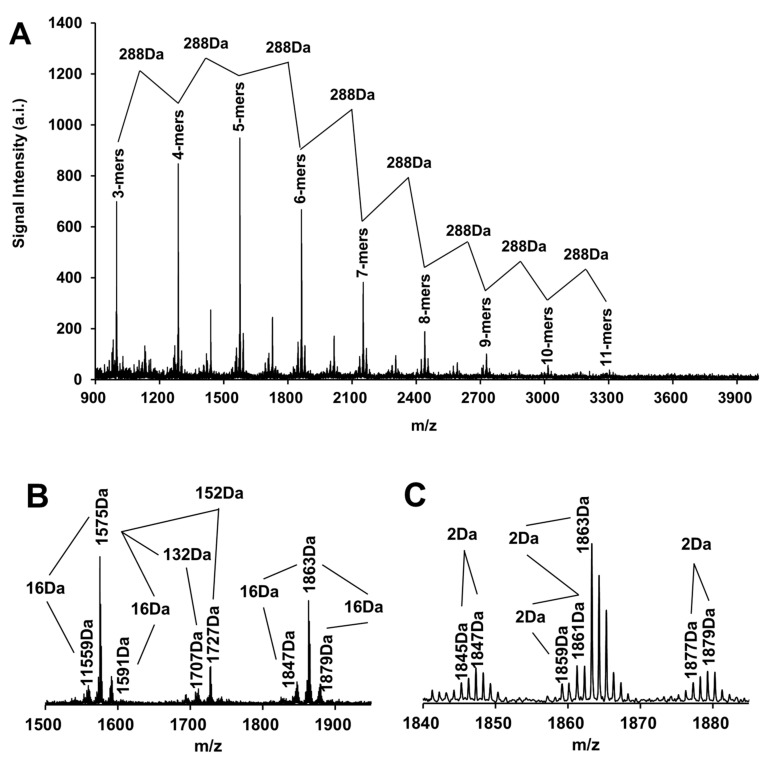

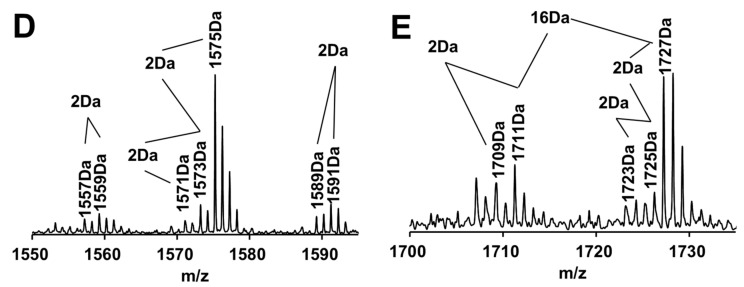
The MALDI-TOF MS spectrum of the 70% acetone eluent of the rhizome of *F. dibotrys* ((**A**) represents the spectrum of the ion peak in the range of *m*/*z* 900–4000, while (**B**–**E**) represent the locally amplified spectrum).

Further, after adequate deionization, the cationic reagent CS^+^ was introduced, and DHB was used as the matrix to obtain a clear MALDI-TOF MS spectrum of proanthocyanidins in the 70% acetone eluent of the rhizome of *F. dibotrys* in reflection mode as shown in Figure 5. According to the previous analysis results, proanthocyanidins in the 70% acetone eluent of the rhizome of *F. dibotrys* were mainly composed of (epi)catechin, (epi)gallocatechin, (epi)catechin gallate, (epi) afzelechin and A-type dimer composed of (epi)catechin monomers, according to the molecular weight of different monomers, the analytical equation for proanthocyanidins in the 70% acetone eluent of the rhizome of *F. dibotrys* was established as follows:

[M + Cs]^+^ = 133 + 2 + 288a + 304b + 272c + 440d − 2e (1)

In Equation (1), *m*/*z* 133 is the atomic mass of CS^+^, *m*/*z* 2 was the atomic mass of the two-terminal hydrogen atoms, a, b, c, d, and e represent the number of (epi)catechin (*m*/*z* 288), (epi)gallocatechin (*m*/*z* 304), (epi)afzelechin (*m*/*z* 272), (epi)catechin gallate (*m*/*z* 440), and A-type linkage respectively [33]. The theoretical [M + Cs]^+^ calculated according to Equation (1) was consistent with the actual detected [M + Cs]^+^, indicating that the established analytical equation was reasonable (Table 9). Based on the comprehensive analysis of the information in Figure 5 and Table 9, the distribution of polymeric proanthocyanidins from trimer (*m*/*z* 999) to undecamer (*m*/*z* 3303) were detected in the 70% acetone eluent of the rhizome of *F. dibotrys*, and the strongest molecular ion peak came from one pentamer (*m*/*z* 1575). The strongest molecular ion peak sequence from trimer to undecamer was *m*/*z* 999–1287–1575–1863–2015–2303–2439–2727–3015–3303 and could be detected in the MALDI-TOF MS spectrum. There was a difference of 288 Da between the ion peak sequence of adjacent molecules, and 288 Da just corresponded to one (epi)catechin monomer. According to Equation (1), the above molecular ion peak sequence corresponded to the proanthocyanidins molecules composed of 3–11 (epi)catechin monomers, respectively, which was consistent with the results of ^13^C NMR analysis and RP-HPLC-ESI-MS analysis of the thiolytic degradation products [33].

In the locally amplified spectrum (Figure 5B), a series of molecular ion peaks with a 132 Da difference from the strongest molecular ion peaks of adjacent polymers were detected. While 132 Da represented the relative atomic mass of one CS^+^ losing one proton, so these two groups of molecular ion peak sequences [M + 2Cs^+^ − H] and [M + Cs^+^ − H] corresponded to the same polymeric proanthocyanidins sequence [33]. Similarly, in the locally amplified spectrum (Figure 5B), a series of molecular ion peaks with a difference of 152 Da were detected between the strongest molecular ion peaks of the adjacent polymers. For example, the molecular ion peak sequence *m*/*z* 1151–1439–1727–2015–2303–2591–2879 was 152 Da different from that of the molecular ion peak sequence *m*/*z* 999–1287–1575–1863–2015–2303–2439–2707 respectively, and 152 Da was the relative molecular weight difference between one (epi)catechin gallate monomer (*m*/*z* 440) and one (epi)catechin monomer (*m*/*z* 288) [33]. In addition, molecular ion peak signals with a difference of 16 Da from the main molecular ion peaks could be detected in the locally amplified spectrum (Figure 5B–E), such as those between *m*/*z* 1559–1575–1591, *m*/*z* 1711–1727, and *m*/*z* 1847–1863–1879, and 16 Da was the atomic mass of one oxygen atom, corresponding to the oxygen atom between (epi)afzelechin and (epi)catechin, or (epi)catechin and (epi)gallocatechin [33]. These results were consistent with the results of RP-HPLC-ESI-MS analysis of thiolytic degradation products, indicating that the presence of (epi)catechin gallate, (epi)gallocatechin, and (epi)afzelechin monomers of proanthocyanidins in the 70% acetone eluent of the rhizome of *F. dibotrys* could be detected by MALDI-TOF MS and could be directly demonstrated in the spectrum.

Two more hydrogen atoms were consumed when an A-type linkage was formed between two monomers by a C-C bond and a C-O-C bond than when a B-type linkage was formed between two monomers by only a C-C bond, while 2 Da corresponded to the atomic mass of two hydrogen atoms. Therefore, the existence of A-type linkage could be intuitively judged by analyzing the presence or absence of molecular ion peaks detected on MALDI-TOF MS spectrum with a 2 Da difference from the main molecular ion peaks [30,31,32,33]. Many molecular ion peaks signals with a 2Da difference from the main molecular ion peaks were detected from the locally amplified spectrum (Figure 5C–E), such as molecular ion peak sequences *m/z* 1557–1559, *m/z* 1571–1573–1575, *m/z* 1589–1591, *m/z* 1845–1847, *m/z* 1859–1861–1863, *m/z* 1877–1879, as well as *m/z* 1709–1711, and *m/z* 1723–1725–1727, indicating that A-type linkages were prevalent in proanthocyanidins molecules of the 70% acetone eluent of the rhizome of *F. dibotrys*. For example, the molecular ion peak sequence *m/z* 1571–1573–1575 represented three pentamers composed of five (epi)catechin monomers with two, one, and zero A-type linkage in their extension units, respectively. Previously, according to the RP-HPLC-ESI-MS analysis results of thiolytic degradation products, it was believed that the A-type linkage of proanthocyanidins in the 70% acetone eluent of the rhizome of *F. dibotrys* only existed in the extension units. Then, MALDI-TOF MS clearly detected the existence of A-type linkages and the number of A-type linkage in different proanthocyanidins molecules, indicating that different analytical methods used in this study could confirm each other, and the comprehensive use of various analytical methods was very helpful for obtaining more detailed structural information of proanthocyanidins [31,32,33].

## 3. Materials and Methods

### 3.1. Materials and Reagents

Seven species of common Polygonaceae plants, including *Fagopyrum dibotrys* [10,13,14], *Fallopia multiflora* [40,41], *Polygonum aviculare* [42,43,44], *Polygonum orientale* [45,46], *Reynoutria japonica* [11], *Rheum officinale* [12], and *Rumex acetosa* [47,48,49,50] were kindly supplied by Bozhou Chinese herbal medicine market in Anhui province (Anhui, China) and identified by Prof. Z.S. Liang, Northwest A&F University. Then the rhizome of *F. dibotrys*, the shoots of *F. dibotrys*, the rhizome of *F. multiflora*, the shoots of *F. multiflora*, the whole plant of *P. aviculare*, the whole plant of *P. orientale*, the rhizome of *R. japonica*, the shoots of *R. japonica*, the rhizome of *R. officinale*, the shoots of *R. officinale*, the rhizome of *R. acetosa* and the shoots of *R. acetosa* were chosen as the experiment materials. Grape seed was selected as the control. All the samples were washed with deionized water, chopped, dried at 45 °C, crushed and sieved through 80 mesh, sealed and kept away from light at −20 °C.

Deuterium dimethyl sulfoxide (DMSO-*d*_6_), benzyl mercaptan, cesium chloride (purity ≥ 99.999%), 2′5-dihydroxybenzoic acid (DHB), Dowex^®^ 50W X8 hydrogen strong acid cation exchange resin (200–400 mesh), chromatographic trifluoroacetic acid (TFA), catechin, epicatechin, gallocatechin, epigallocatechin, catechin gallate, epicatechin gallate, grape seed proanthocyanidins reference standard (GsPs), *Saccharomyces cerevisiae α*-glucosidase (type I, from *Saccharomyces cerevisiae*), porcine pancreas *α*-amylase (from porcine pancreas), human saliva *α*-amylase (from human saliva), 2,2-diazo-bis(3-ethyl-benzothiazole-6-sulfonic acid) diammonium salt (ABTS), 6-hydroxy-2,5,7,8-tetramethylchromo-2-carboxylic acid (Trolox), 2,4,6-tripyridinyl-1,3,5-triazine (TPTZ), Vitamin C, 1,1-diphenyl-2-picrylhydrazyl (DPPH), Neocuproine hydrochloride monohydrate (NHCM), acarbose, and 4-nitrophenyl-*α*-D-glucopyranoside (*p*-NPG) were purchased from Sigma-Aldrich Chemical Co. (St. Louis, MO, USA). Sephadex LH-20 was purchased from GE Healthcare Bio-Sciences AB (Uppsala, Sweden). 2-choro-4-nitrophenyl-*α*-galactosyl-maltoside (Gal-G*_2_*-*α*-CNP) was purchased from Toyobo Co., Ltd., (Osaka, Japan). Methanol, acetone, and acetonitrile, all with chromatographic purity, were purchased from Tedia (Fairfield, OH, USA). Potassium bromide and methanol, both of pure spectrum grade, were from Aladdin Reagents Co., Ltd. (Shanghai, China). Other reagents, including ferric chloride, potassium persulfate, copper sulfate, acetic acid, sodium acetate, petroleum ether, potassium dihydrogen phosphate, and dipotassium hydrogen phosphate, were of analytical grade and from Sinopharm Chemical Reagent Co., Ltd. (Shanghai, China). The water used in this study was deionized water.

### 3.2. Sample Preparation

#### 3.2.1. Preparation of Plant Crude Extracts

The powders of the seven species of Polygonaceae plant materials and grape seed were mixed with 85% methanol (volume fraction, the same below) at a solid-liquid ratio of 1:20 (g/L), and then stood for 12 h separately. Then the mixtures were extracted at 45 °C with 500 W ultrasonic power for 40 min and then filtered. The filter residues were successively extracted with 70% methanol and 60% ethanol in accordance with the above-mentioned parameters, and the filtrates were concentrated under reduced pressure at 45 °C and 0.09 MPa to recover the solvent. The concentrated solution of each sample was combined, then degreased with petroleum ether 5 times, and then lyophilized to obtain the crude extract of each sample, which were sealed at −80 °C and kept out of light [13].

#### 3.2.2. Isolation and Purification of Proanthocyanidins

The crude extract of the rhizome of *F. dibotrys* was prepared from the rhizome of *F. dibotrys* powder according to the method described in Section 3.2.1. Seven and a half grams of the crude extract was fully dissolved in 50 mL of 50% methanol and then filtered by 0.45 μm microporous membrane, and the filtrate was then enriched and purified by Sephadex LH-20. Sephadex LH-20 was fully swelled with 50% methanol and then loaded into a glass chromatography column with a volume of 1.2 L. The column was first balanced with 5 L of 50% degassed methanol, then the filtrate was carefully loaded, and the piston of the column was closed for adsorption for 6 h. First, the column was eluted by 5 L of 50% degassed methanol at a rate of 1.0 mL/min; the eluent was collected and concentrated under reduced pressure at 45 °C and 0.09 MPa, and then lyophilized to obtain the 50% methanol eluent. Then, the column was subsequently eluted by 2.5 L of 70% degassed acetone at a rate of 3.0 mL/min; the eluent was collected and concentrated under reduced pressure at 45 °C and 0.09 MPa and then lyophilized to obtain the 70% acetone eluent [33].

#### 3.2.3. Preparation of Sample Solution

The crude extracts of the seven species of Polygonaceae plant materials and grape seed were fully dissolved in 10% DMSO to obtain a solution equivalent to 10 mg DW/mL. Then all the solutions were filtered by a 0.45 μm microporous membrane and diluted into solutions equivalent to 10 μg DW/mL with NaH_2_PO_4_–Na_2_HPO_4_ buffer (100 mM, pH = 6.86) to determine their inhibitory activity against *S. cerevisiae α*-glucosidase; and diluted into solutions equivalent to 100 μg DW/mL with NaH_2_PO_4_–Na_2_HPO_4_ buffer (100 mM, pH = 6.86, containing 6.7 mM NaCl) to determine their inhibitory activities against porcine pancreatic *α*-amylase and human salivary *α*-amylase [4,51]. Crude extract, the 50% methanol eluent, and the 70% acetone eluent of the rhizome of *F. dibotrys* were fully dissolved in 10% DMSO to obtain solutions equivalent to 10 mg DW/mL, filtrated, and then diluted with the corresponding buffer to their most suitable concentration of each to determine their half inhibitory concentrations (IC_50_ values) against *S. cerevisiae α*-glucosidase, porcine pancreatic *α*-amylase, and human salivary *α*-amylase.

The crude extracts of the seven species of Polygonaceae plant materials and grape seed were fully dissolved in 80% methanol to obtain solutions equivalent to 10 mg DW/mL. Then all the solutions were filtered by a 0.45 μm microporous membrane and diluted into solutions equivalent to 500 μg DW/mL with methanol to determine the DPPH and ABTS free radical scavenging rates of each sample and diluted with methanol to their most suitable concentration to determine their total proanthocyanidins content (TOPCs), Ferric ion reducing antioxidant power (FRAP) and Cupric ion reducing power (CUPRAC). Crude extract, the 50% methanol eluent, and the 70% acetone eluent of the rhizome of *F. dibotrys* were fully dissolved in 80% methanol to obtain solutions equivalent to 10 mg DW/mL, filtrated, and then diluted with methanol to their most suitable concentration of each to determine their TOPCs, their half scavenging concentrations (EC_50_ values) against DPPH and ABTS free radical, and their FRAP and CUPRAC values.

### 3.3. Determination of Total Proanthocyanidins Content (TOPCs)

GsPs was selected as the control, 200 μL of GsPs solution of different concentrations was added into a glass test tube, followed by 7500 μL of HCl-n-butanol solution (with a volume ratio of 5:95) and 100 μL of 2% ammonium ferric sulfate solution (2 M HCl as the solvent). After fully mixing, the test tube was placed in boiling water for 75 min and then cooled by ice bath immediately. When the temperature was restored to room temperature, the absorbance value at 550 nm (A_550_) was measured on a UV-1700 UV-Visible Spectrophotometer (Shimadzu, Kyoto, Japan) with distilled water as blank. The standard curve was drawn with the concentration of GsPs as the *X*-axis and the absorbance as the *Y*-axis, and the linear regression equation *Y* = 0.9933*X* − 0.0045 (*R*^2^ = 0.9998) was obtained, with the linear range of 0.00–1.00 mg/mL. The solution of each sample was diluted with methanol to their appropriate concentration so that the absorbance value at 550 nm was between 0.200 and 0.800. The A_550_ value of each sample was determined respectively, and the TOPCs of each sample were calculated according to the linear regression equation and expressed as the equivalent value of each sample to GsPs (mg GsPs/g DW) [36,37].

### 3.4. Antioxidant Assay

#### 3.4.1. DPPH Assay

DPPH free radical solution was prepared with methanol with a concentration of 100 μM. One hundred microliters of the sample solution was thoroughly mixed with 3000 μL of DPPH free radical solution, and the absorbance value (A_517_) at 517 nm was determined on a UV-1700 UV-Visible Spectrophotometer after reaction in the dark place at room temperature for 30 min, with methanol as blank. DPPH free radical scavenging rates of samples with different concentrations were calculated according to Formula (2):

DPPH free radical scavenging rate % = (A_0_ − A_i_)/A_0_ × 100 (2)

In Formula (2), A_0_ and A_i_ respectively represent the absorbance values of blank samples (methanol) and sample solutions of different concentrations after reacting with DPPH free radical solution. The DPPH free radical scavenging activities of the seven species of Polygonaceae plant materials and grape seed were expressed as the DPPH scavenging rates of each sample at the concentration of 500 μg DW/mL. According to the pre-test results, sample solutions of crude extract, 50% methanol eluent, and 70% acetone eluent of the rhizome of *F. dibotrys* were diluted with methanol into eight appropriate concentration gradients of each so that the DPPH free radical scavenging rate of each sample under the maximum concentration was ≥80%. The DPPH free radical scavenging activity of each sample was represented by the half scavenging concentration of DPPH free radical (EC_50_ value) of the sample. GsPs and Vc were selected as the control substances, and the smaller EC_50_ value meant the stronger DPPH free radical scavenging activity [31,32,33,52].

#### 3.4.2. ABTS Assay

ABTS free radical was prepared by the reaction of ABTS aqueous solution with potassium persulfate, and ABTS free radical was diluted with ethanol to a solution with an absorbance value of about 0.700 ± 0.020 at 734 nm [40]. The sample solution of 100 μL was thoroughly mixed with ABTS free radical solution of 3000 μL, and the absorbance value (A_734_) at 734 nm was determined on a UV-1700 UV-Visible Spectrophotometer after reaction in the dark place at room temperature for 30 min, with methanol as blank. ABTS free radical scavenging rates of samples with different concentrations were calculated according to Formula (3):

ABTS free radical scavenging rate % = (A_0_ − A_i_)/A_0_ × 100 (3)

In Formula (3), A_0_ and A_i_ respectively represent the absorbance values of blank samples (methanol) and sample solutions of different concentrations after reacting with ABTS free radical solutions. The ABTS free radical scavenging activities of the seven species of Polygonaceae plant materials and grape seed were expressed as the ABTS scavenging rates of each sample at the concentration of 500 μg DW/mL. According to the pre-test results, sample solutions of crude extract, 50% methanol eluent, and 70% acetone eluent of the rhizome of *F. dibotrys* were diluted with methanol into eight appropriate concentration gradients of each so that the ABTS free radical scavenging rate of each sample under the maximum concentration was ≥80%. The ABTS free radical scavenging activity of each sample was represented by the half scavenging concentration of ABTS free radical (EC_50_ value) of the sample. GsPs and Vc were selected as the control substances, and the smaller EC_50_ value meant the stronger ABTS free radical scavenging activity [53].

#### 3.4.3. FRAP Assay

The FRAP reagent was prepared by mixing acetic acid-sodium acetate buffer (300 mM, pH = 3.6), TPTZ solution (10 mM, 40 mM HCl as the solvent) and FeCl_3_ aqueous solution (20 mM) in a 10:1:1 volume ratio [42]. One hundred microliters of Vc solution of different concentrations were added into a test tube, followed by 3000 μL of FRAP reagent, and thoroughly mixed. After reaction at room temperature for 30 min, the absorbance value (A_593_) of the solution at 593 nm was determined on a UV-1700 UV-Visible Spectrophotometer with distilled water as blank. Taking the concentration of Vc solution as the *X*-axis and the absorbance as the *Y*-axis, the standard curve was drawn, and the linear regression equation was *Y* = 14.3510*X* + 0.0670 (*R*^2^ = 0.9996), with a linear range of 0.00–0.15 mg/mL. The solution of each sample was diluted with methanol to an appropriate concentration so that the absorbance value at 593 nm was between 0.200 and 0.800. The A_593_ value of each sample was determined respectively, and the FRAP value of each sample was calculated according to the linear regression equation and expressed as the equivalent value of each sample to Vc (mg Vc/g equivalent) [31,54].

#### 3.4.4. CUPRAC Assay

One hundred microliters of Trolox solution of different concentrations were added into a test tube, and then 1000 μL of 5 mM CuSO_4_ aqueous solution, 1000 μL of 1 M aceto-ammonium acetate buffer, 1000 μL of 3.75 mM NHCM ethanol solution, and 1000 μL of distilled water were added into the same test tube successively and thoroughly mixed. After reaction at room temperature for 30 min, the absorbance value (A_450_) of the solution at 450 nm was determined on a UV-1700 UV-Visible Spectrophotometer with distilled water as blank. Taking the concentration of Trolox solution as the *X*-axis and the absorbance as the *Y*-axis, the standard curve was drawn, and the linear regression equation was *Y* = 1.7501*X* + 0.0032 (*R*^2^ = 0.9992), with a linear range of 0.00–0.40 mg/mL. The solution of each sample was diluted with methanol to an appropriate concentration so that the absorbance value at 450 nm was between 0.200 and 0.800. The A_450_ value of each sample was determined respectively, and the CUPRAC value of each sample was calculated according to the linear regression equation and expressed as the equivalent value of each sample to Trolox (mg Trolox/g equivalent) [55].

### 3.5. Antidiabetic Assay

*p*-NPG was used as a substrate for *S. cerevisiae α*-glucosidase, and Gal-G_2_-*α*-CNP was used as a substrate for porcine pancreatic *α*-amylase and human salivary *α*-amylase. NaH_2_PO_4_-Na_2_HPO_4_ buffer solution (100 mM, pH = 6.86) was prepared, and the buffer solution was used as the solvent to prepare the *S. cerevisiae α*-glucosidase solution with the concentration of 100 U/L and substrate solution with the concentration of 5 mM, respectively. NaH_2_PO_4_–Na_2_HPO_4_ buffer (50 mM, pH = 6.86, containing 6.7 mM NaCl) was prepared, and the buffer was used as a solvent to prepare the porcine pancreatic *α*-amylase with the concentration of 1250 U/L and human salivary *α*-amylase with the concentration of 2500 U/L and substrate solution with the concentration of 5 mM, respectively. 50 μL of buffer solution, 50 μL of enzyme solution, and 50 μL of sample solution were successively added into each well of a 96-well plate and thoroughly mixed. After incubated at 37 °C for 10 min, 50 μL of substrate solution was added into each well and thoroughly mixed, and the increase in absorbance at 405 nm (Δ_405_) within 15 min was recorded on a SpectraMax M2 Microplate Reader (Molecular Devices, California, USA). The enzyme inhibition rates of samples with different concentrations were calculated according to Formula (4):

enzyme inhibition rate % = (Δ_0_ − Δ_i_)/Δ_0_ × 100 (4)

In Formula (4), Δ_0_ and Δ_i_ respectively represent the increase in absorbance at 405 nm after the blank sample (buffer) and sample solutions with different concentrations interacted with the enzyme-substrate system. The inhibitory activities of the seven species of Polygonaceae plant materials and grape seed against *S. cerevisiae α*-glucosidase were expressed as the inhibition rates of each sample at the concentration of 10 μg DW/mL, and the inhibitory activities of the seven species of Polygonaceae plant materials and grape seed against porcine pancreatic *α*-amylase and human salivary *α*-amylase were expressed as the inhibition rates of each sample at the concentration of 100 μg DW/mL. According to the pre-test results, sample solutions of crude extract, the 50% methanol eluent, and the 70% acetone eluent of the rhizome of *F. dibotrys* were diluted with buffer solution into eight appropriate concentration gradients of each so that the enzymatic inhibition rate of each sample under the maximum concentration was ≥80%. The enzymatic inhibitory activity of each sample was represented by the half inhibition concentration of each enzyme (IC_50_ value) of the sample. GsPs and acarbose were selected as the control substances, and the smaller IC_50_ value meant the stronger enzymatic inhibitory activity [2,3,4,5,51].

### 3.6. Structure Analysis of Proanthocyanidins

#### 3.6.1. UV-Vis Analysis

The 70% acetone eluent of the rhizome of *F. dibotrys* and GsPs were prepared with methanol at a concentration of 50 μg/mL, respectively. The absorption spectrum of the samples was drawn at 200–400 nm after blank scanning with methanol on a UV-1700 UV-visible spectrophotometer to obtain the UV spectrum of the samples [4]. Then the 70% acetone eluent of the rhizome of *F. dibotrys* and GsPs were prepared with methanol at a concentration of 1.00 mg/mL, respectively. After color development according to the method described in Section 3.3, the absorption spectrum of the samples was drawn at 400–800 nm after blank scanning with distilled water on a UV-1700 UV-visible spectrophotometer to obtain the visible spectrum of the samples [36].

#### 3.6.2. FT-IR Analysis

The 70% acetone eluent of the rhizome of *F. dibotrys* and GsPs were mixed with potassium bromide after fully drying and pressed into tablets. The FT-IR spectrum in the wave number range of 4000–400 cm^−1^ were plotted by a Nicolet IS 5 FT-IR Spectrometer (Thermo Scientific, San Jose, CA, USA) with potassium bromide as blank scanning. The scanning resolution and scanning times were set to 4 cm^−1^ and 32 times [4].

#### 3.6.3. ^13^C NMR Analysis

One hundred and twenty-five micrograms of the 70% acetone eluent of the rhizome of *F. dibotrys* and GsPs were dissolved in 600 μL of DMSO-*d_6_* separately, and the ^13^C NMR spectrum was collected by a Bruker nuclear magnetic resonance spectrometer (Avance III 500, Bruker, Karlsruhe, Germany). The scanning frequency, pulse angle, and delay time were 126 MHz, 45°, and 3 s, respectively [32].

#### 3.6.4. Benzyl Mercaptan Degradation of Proanthocyanidins and RP-HPLC-ESI-MS Analysis of Thiolytic Degradation Products

Five micrograms of the 70% acetone eluent of the rhizome of *F. dibotrys* were dissolved in 1 mL of methanol and fully mixed with 1mL of 3.3% (*v/v*) of HCl-methanol solution and 2 mL of 5% (*v/v*) of benzyl mercaptan-methanol solution. The degradation products were obtained by reaction at 40 °C for 30 min and filtered by 0.22 μm microporous membrane for analysis [33].

The RP-HPLC analysis was performed on a Waters 1525 binary high-performance liquid chromatograph system with Waters XBridge Beh Shield RP18 (130 Å, 5 μm, 4.6 mm × 250 mm) column and Waters 2996 photodiode array detector (Waters, Milford, MA, USA). Mobile phase A and B were acetonitrile and 0.5% (*v/v*) TFA aqueous solution, respectively, and the elution gradient was 0–45 min, 12%–80% A; 45–50 min, 80%–12% A. The injection volume was 20 μL, the flow rate was 1.0 mL/min, the column temperature was 25 °C, and the detection wavelength was 280 nm.

The ESI-MS analysis was performed on an LTQ-XL linear ion trap mass spectrometer (Thermo Scientific, San Jose, CA, USA) with a split ratio of 1:3, a capillary temperature of 400 °C, an electrospray voltage of 4.50 kV, a sheath gas pressure of 50 psi, an auxiliary gas pressure of 10 psi, an intra source collision-induced disintegration energy of 10 V and collision energy of 50 V, and a negative ion scanning range of *m/z* 100–800.

The thiolytic degradation products of the 70% acetone eluent of the rhizome of *F. dibotrys* were identified by the chromatographic retention time, and the mass spectrum data and the mean degree of polymerization (mDP) were calculated according to formula (4) proposed by Zhou et al. [33]:

mDP = 1 + total peak area of extension units/total peak area of terminal units(5)

#### 3.6.5. MALDI-TOF MS Analysis

According to the method of Zhou et al. [33], the 70% acetone eluent of the rhizome of *F. dibotrys* was prepared with 30% (*v/v*) acetone-water solution into a 10 mg/mL solution and deionized with Dowex^®^ 50W X8 resin. After that, the sample was fully mixed with CsCl (1.52 mg/mL aqueous solution) and DHB (10 mg/mL, 30% (*v/v*) acetone-water solution as the solvent) at a volume ratio of 1:1:6. Then 1.0 μL of the mixture were sampled onto the target and dried at room temperature for analysis by a MALDI-TOF MS analyzer (ABI 4700 MALDI-TOF/TOF, Waltham, MA, USA). The target was calibrated using external standards Angiotensin II (1046.5 Da), Bombesin (1619.8 Da), Acthclip 18–39 (2465.2 Da), and Somatostatin 28 (3147.47 Da) before analysis. The analytical parameters were as follows: the wavelength of N_2_ laser was 337 nm, the pulse width was 3 ns, the acceleration and reflection voltage of reflection mode analysis were 20.0 kV and 23.0 kV, respectively, the optimal mass resolution was 2000 Da, the positive ion scanning range was *m/z* 900–4000 Da, and the number of scanning was 200–500 times.

### 3.7. Statistical Analysis

All data measurements in this study were repeated 3 times, and the results were expressed as Mean ± SD. One-way analysis of variance (One-way ANVOA) and Student’s *t* test were performed using the SPSS22.0 software (SPSS Inc., Chicago, IL, USA), and *p* < 0.05 was considered to be statistically significant.

## 4. Conclusions

The results of this study indicated that proanthocyanidins were the main antioxidant and antidiabetic active substances in the rhizome of *F. dibotrys*, and proanthocyanidins purified from the rhizome of *F. dibotrys* showed similar antioxidant activity to GsPs and stronger antidiabetic activity than GsPs. Proanthocyanidins were identified from the 70% acetone eluent in the rhizome of *F. dibotrys*, and their structures were characterized with catechin and epicatechin accounted for more than 90% of all monomers and the mDP was about 5.02 ± 0.21. This study is expected to provide guidance and reference for the development of *F. dibotrys* as a potential antioxidant and hypoglycemic product. However, for further elucidate the mechanism of antioxidant and antidiabetes of *F. dibotrys*, and to better and more safely exploit and utilize the plant resources of *F. dibotrys*, enough cells, animals, and clinical trials are essential.

## Figures and Tables

**Table 1:** The TOPCs of seven plant materials of the Polygonaceae family and grape seed.

Category	Plant Sample	TOPCs (mg GsPs/g DW)
Control	Grape seed	75.24 ± 0.98 ^b^
A	Rhizome of *F. dibotrys*	82.14 ± 0.51 ^a^
B	Shoots of *F. dibotrys*	12.66 ± 0.44 ^e^
C	Rhizome of *F. multiflora*	4.18 ± 0.33 ^g^
D	Shoots of *F. multiflora*	0.94 ± 0.42 ^ij^
E	Whole plant of *P. aviculare*	0.82 ± 0.18 ^j^
F	Whole plant of *P. orientale*	0.35 ± 0.09 ^k^
G	Rhizome of *R. japonica*	13.22 ± 0.44 ^d^
H	Shoots of *R. japonica*	0.50 ± 0.27 ^k^
I	Rhizome of *R. officinale*	22.19 ± 0.35 ^c^
J	Shoots of *R. officinale*	1.34 ± 0.50 ^i^
K	Rhizome of *R. acetosa*	8.28 ± 0.14 ^f^
L	Shoots of *R. acetosa*	3.82 ± 0.10 ^h^

Different lowercase letters in each column indicate significant differences at the *p* < 0.05 level.

**Table 2:** The antioxidant activity of DPPH, ABTS, FRAP, and CUPRAC of seven plant materials of the Polygonaceae family and grape seed.

Category	DPPH Scavenging (%)	ABTS Scavenging (%)	FRAP/mg Vc/g Equivalent	CUPRAC/mg Trolox/g Equivalent
Control	67.68 ± 0.25 ^b^	92.57 ± 0.74 ^a^	115.95 ± 0.85 ^a^	208.89 ± 2.24 ^b^
A	72.11 ± 0.42 ^a^	93.20 ± 0.29 ^a^	103.12 ± 1.50 ^b^	215.23 ± 0.59 ^a^
B	22.48 ± 0.19 ^g^	28.75 ± 1.36 ^h^	25.46 ± 0.38 ^g^	48.45 ± 1.32 ^g^
C	32.28 ± 0.33 ^f^	39.25 ± 0.28 ^f^	68.84 ± 0.86 ^e^	156.20 ± 3.25 ^c^
D	9.12 ± 0.48 ^k^	22.48 ± 0.30 ^i^	6.58 ± 0.32 ^k^	14.23 ± 0.85 ^m^
E	6.75 ± 0.50 ^m^	11.34 ± 0.25 ^k^	11.13 ± 0.45 ^i^	26.04 ± 1.20 ^i^
F	8.49 ± 0.24 ^l^	20.28 ± 0.19 ^j^	16.92 ± 0.32 ^h^	37.67 ± 0.47 ^h^
G	44.62 ± 0.36 ^d^	58.69 ± 0.40 ^c^	71.42 ± 0.29 ^d^	128.29 ± 2.19 ^e^
H	18.23 ± 0.19 ^h^	41.08 ± 0.28 ^e^	9.79 ± 0.08 ^j^	22.14 ± 0.56 ^l^
I	36.50 ± 0.42 ^e^	58.17 ± 0.13 ^d^	76.60 ± 0.94 ^c^	131.25 ± 0.47 ^d^
J	14.25 ± 0.28 ^i^	31.20 ± 0.96 ^g^	11.49 ± 0.40 ^i^	24.30 ± 0.32 ^j^
K	45.40 ± 0.63 ^c^	67.25 ± 1.27 ^b^	53.49 ± 0.34 ^f^	118.21 ± 0.18 ^f^
L	12.14 ± 0.19 ^j^	20.38 ± 0.50 ^j^	10.16 ± 0.69 ^j^	23.28 ± 0.79 ^k^

Different lowercase letters in each column indicate significant differences at the *p* < 0.05 level.

**Table 3:** The inhibitory activity of seven plant materials of the Polygonaceae family and grape seed against *S. cerevisiae α*-glucosidase, porcine pancreatic *α*-amylase, and human salivary α-amylase.

Category	*S. cerevisiae* α-Glucosidase Inhibition (%)	Porcine Pancreatic α-Amylase Inhibition (%)	Human Salivary α-Amylase Inhibition (%)
Control	96.29 ± 1.32 ^a^	98.75 ± 0.49 ^a^	74.28 ± 2.21 ^b^
A	83.17 ± 0.88 ^b^	96.27 ± 1.28 ^b^	85.76 ± 0.48 ^a^
B	14.59 ± 0.24 ^g^	10.23 ± 0.36 ^h^	5.26 ± 0.34 ^gh^
C	29.54 ± 1.44 ^e^	32.18 ± 0.27 ^d^	17.64 ± 1.42 ^d^
D	8.79 ± 0.55 ^i^	10.20 ± 1.38 ^h^	4.78 ± 0.59 ^h^
E	2.46 ± 1.82 ^k^	5.45 ± 0.59 ^j^	1.65 ± 0.16 ^j^
F	4.18 ± 1.33 ^k^	7.60 ± 0.72 ^i^	3.44 ± 0.84 ^i^
G	37.94 ± 3.16 ^d^	30.28 ± 0.46 ^e^	16.29 ± 0.23 ^d^
H	6.50 ± 1.22 ^j^	8.51 ± 2.28 ^hi^	3.51 ± 0.96 ^i^
I	60.29 ± 1.44 ^c^	45.46 ± 1.59 ^c^	40.24 ± 2.10 ^c^
J	8.68 ± 0.13 ^i^	12.49 ± 0.33 ^g^	7.57 ± 0.96 ^f^
K	24.29 ± 2.44 ^f^	18.35 ± 2.12 ^f^	11.39 ± 1.05 ^e^
L	12.52 ± 1.67 ^h^	10.49 ± 0.56 ^h^	5.62 ± 0.32 ^g^

Different lowercase letters in each column indicate significant differences at the *p* < 0.05 level.

**Table 4:** The Pearson correlation coefficient between TOPCs, and antioxidant and antidiabetic activity.

Pearson Correlation Coefficient	TOPCs	DPPH	ABTS	FRAP	CUPRAC	α-glu	PPA
DPPH	0.8765 ^**^						
ABTS	0.8565 ^**^	0.9785 ^**^					
FRAP	0.8331 ^**^	0.9487 ^**^	0.9092 ^**^				
CUPRAC	0.8190 ^**^	0.9495 ^**^	0.8995 ^**^	0.9880 ^**^			
α-glu	0.9295 ^**^	0.9217 ^**^	0.9091 ^**^	0.9512 ^**^	0.9177 ^**^		
PPA	0.9694 ^**^	0.9080 ^**^	0.8905 ^**^	0.9163 ^**^	0.9020 ^**^	0.9774 ^**^	
HSA	0.9753 ^**^	0.8863 ^**^	0.8763 ^**^	0.8829 ^**^	0.8706 ^**^	0.9641 ^**^	0.9898 ^**^

** indicates significant correlations at the *p* < 0.01 level.

**Table 5:** Comparison of TOPCs of the 50% methanol eluent, crude extract and the 70% acetone eluent of rhizome of *F. dibotrys.*

Category	TOPCs (mg GsPs/g DW)
50% methanol eluent	15.39 ± 0.25 ^c^
Crude extract	245.24 ± 4.71 ^b^
70% acetone eluent	1223.15 ± 20.64 ^a^

Different lowercase letters in each column indicate significant differences at the *p* < 0.05 level.

**Table 6:** The antioxidant activities of DPPH, ABTS, FRAP and CUPRAC of the 50% methanol eluent, crude extract and the 70% acetone eluent of rhizome of *F. dibotrys*, GsPs, Vc and Trolox.

Category	EC_50_/DPPH (µg/mL)	EC_50_/ABTS (µg/mL)	FRAP/mg Vc/g Equivalent	CUPRAC/mg Trolox/g Equivalent
50% methanol eluent	514.58 ± 4.42 ^a^	288.78 ± 2.36 ^a^	113.20 ± 1.47 ^e^	323.28 ± 3.80 ^d^
Crude extract	239.46 ± 2.18 ^b^	184.3 ± 1.96 ^b^	278.22 ± 2.12 ^d^	581.74 ± 5.25 ^c^
70% acetone eluent	82.00 ± 0.63 ^e^	54.78 ± 0.45 ^d^	674.52 ± 4.64 ^c^	1999.71 ± 14.31 ^a^
GsPs	86.25 ± 0.77 ^d^	54.47 ± 0.35 ^d^	728.55 ± 4.62 ^b^	1996.59 ± 18.45 ^a^
Vc	128.33 ± 0.75 ^c^	82.53 ± 0.96 ^c^	1000.00 ± 11.45 ^a^	-
Trolox	-	-	-	1000.00 ± 16.86 ^b^

Different lowercase letters in each column indicate significant differences at the *p* < 0.05 level.

**Table 7:** The inhibitory activities of the 50% methanol eluent, crude extract and the 70% acetone eluent of rhizome of *F. dibotrys*, GsPs and acarbose against *S. cerevisiae α*-glucosidase, porcine pancreatic *α*-amylase and human salivary *α*-amylase.

Category	IC_50_/*S. cerevisiae* α-Glucosidase (µg/mL)	IC_50_/Porcine Pancreatic α-Amylase (µg/mL)	IC_50_/Human Salivary α-Amylase (µg/mL)
50% methanol eluent	144.36 ± 7.53 ^b^	267.29 ± 6.28 ^a^	428.50 ± 5.18 ^a^
Crude extract	6.54 ± 1.36 ^c^	36.51 ± 0.87 ^b^	58.39 ± 1.49 ^b^
70% acetone eluent	1.28 ± 0.04 ^e^	4.18 ± 0.12 ^d^	7.67 ± 0.18 ^d^
GsPs	1.51 ± 0.02 ^d^	4.55 ± 0.08 ^c^	9.01 ± 0.24 ^c^
Acarbose	244.30 ± 5.25 ^a^	0.45 ± 0.03 ^e^	1.26 ± 0.12 ^e^

Different lowercase letters in each column indicate significant differences at the *p* < 0.05 level.

**Table 8:** Analysis results of benzyl mercaptan degradation products of the 70% acetone eluent of the rhizome of *F. dibotrys* by RP-HPLC.

Category	Peak No.	Sort	Value
Terminal Units (%)	1	C	11.69 ± 0.46
	2	EC	6.83 ± 1.03
	3	ECG	1.41 ± 0.53
Extension Units (%)	4	GC	2.72 ± 0.17
	5	EGC	2.30 ± 0.22
	6	C	6.46 ± 1.93
	7	EC	64.41 ± 3.12
	8	(E) CG	2.59 ± 0.53
	9	A-type dimer	0.78 ± 0.08
	10	(E) AF	0.81 ± 0.24
Spatial configuration (%)	2′3-*cis* configuration		79.13 ± 0.85
	2′3-*trans* configuration		20.87 ± 0.85
Galloyl group (%)			4.00 ± 0.53
A-type linkage (%)			0.78 ± 0.08
mDP			5.02 ± 0.21

C, EC, ECG, GC, EGC, (E)CG, and (E)AF were short for catechin, epicatechin, epicatechin gallate, gallocatechin, epigallocatechin, (epi)catechin gallate, and (epi)afzelechin, respectively.

**Table 9:** MALDI-TOF MS analysis results of the 70% acetone eluent of the rhizome of *F. dibotrys*.

No.	DP	(E)C	(E)GC	(E)AF	(E)CG	A-Type	Cal [M + Cs]^+^	Observed [M + Cs]^+^
1	3-mers	2	0	1	0	2	979	978.9893
2		2	0	1	0	1	981	981.0812
3		2	0	1	0	0	983	983.0591
4		3	0	0	0	2	995	995.0164
5		3	0	0	0	1	997	997.0663
6		3	0	0	0	0	999	999.0757
7		1	0	1	1	0	1135	1135.2317
8		2	0	0	1	1	1149	1149.1038
9		2	0	0	1	0	1151	1151.1265
10	4-mers	3	0	1	0	1	1269	1269.1731
11		3	0	1	0	0	1271	1271.1989
12		4	0	0	0	2	1283	1283.0939
13		4	0	0	0	1	1285	1285.1801
14		4	0	0	0	0	1287	1287.1881
15		3	1	0	0	2	1299	1299.1941
16		3	1	0	0	1	1301	1301.1884
17		3	1	0	0	0	1303	1303.1790
18		2	0	1	1	0	1423	1423.2411
19		3	0	0	1	2	1435	1435.2147
20		3	0	0	1	1	1437	1437.1962
21		3	0	0	1	0	1439	1439.1993
22	5-mers	4	0	1	0	1	1557	1557.2426
23		4	0	1	0	0	1559	1559.2617
24		5	0	0	0	2	1571	1571.1364
25		5	0	0	0	1	1573	1573.2393
26		5	0	0	0	0	1575	1575.2474
27		4	1	0	0	2	1587	1587.2273
28		4	1	0	0	1	1589	1589.2378
29		4	1	0	0	0	1591	1591.2358
30		3	0	1	1	1	1709	1709.2579
31		3	0	1	1	0	1711	1711.2736
32		4	0	0	1	2	1723	1723.2308
33		4	0	0	1	1	1725	1725.2627
34		4	0	0	1	0	1727	1727.2469
35	6-mers	5	0	1	0	1	1845	1845.2870
36		5	0	1	0	0	1847	1847.2938
37		6	0	0	0	2	1859	1859.1938
38		6	0	0	0	1	1861	1861.2612
39		6	0	0	0	0	1863	1863.2898
40		5	1	0	0	1	1877	1877.2839
41		5	1	0	0	0	1879	1879.2777
42		3	0	2	1	1	1981	1981.3029
43		3	0	2	1	0	1983	1983.3977
44		4	0	1	1	1	1997	1997.1848
45		4	0	1	1	0	1999	1999.3131
46		5	0	0	1	2	2011	2011.2957
47		5	0	0	1	1	2013	2013.3037
48		5	0	0	1	0	2015	2015.2927
49	7-mers	6	0	1	0	1	2133	2133.3162
50		6	0	1	0	0	2135	2135.3201
51		7	0	0	0	2	2147	2147.2498
52		7	0	0	0	1	2149	2149.3015
53		7	0	0	0	0	2151	2151.3225
54		6	1	0	0	1	2165	2165.3149
55		6	1	0	0	0	2167	2167.3062
56		5	0	1	1	0	2287	2287.3433
57		6	0	0	1	1	2301	2301.3628
58		6	0	0	1	0	2303	2303.3269
59	8-mers	7	0	1	0	0	2423	2423.3477
60		8	0	0	0	2	2435	2435.3259
61		8	0	0	0	1	2437	2437.3362
62		8	0	0	0	0	2439	2439.3589
63		7	1	0	0	0	2455	2455.3499
64		7	0	0	1	0	2591	2591.3704
65	9-mers	8	0	1	0	0	2711	2711.3899
66		9	0	0	0	0	2727	2727.3938
67		8	1	0	0	0	2743	2743.3208
68		8	0	0	1	0	2879	2879.4021
69	10-mers	9	0	1	0	0	2999	2999.4319
70		10	0	0	0	0	3015	3015.4089
71		9	1	0	0	0	3031	3031.4106
72	11-mers	11	0	0	0	0	3303	3303.4424

(E)C, (E)GC, (E)AF, (E)CG, A-type and Cal [M + Cs]^+^ were short for (epi)catechin, (epi)gallocatechin, (epi)afzelechin, (epi)catechin gallate, A-type linkage, and calculated [M + Cs]^+^, respectively.

## Data Availability

Not applicable.

## Supplementary Materials

Figure S1: The Chemical structure of typical flavane-3-ol monomers. Table S1: The abbreviations in this article. A brief introduction of the seven Polygonaceae plants used in this article is also available in the Supplementary Materials.

## Sample Availability

The proanthocyanidins purified from the rhizome of *F. dibotrys* are available from the authors.
