# Supplementary material for: Antioxidant and Antidiabetic Activity of Proanthocyanidins from Fagopyrum dibotrys
Source: Molecules. 2021 Apr 21;26(9):2417. doi: 10.3390/molecules26092417 (PMC8122523; doi:10.3390/molecules26092417)
Supplement: Supplementary file 1 [file molecules-26-02417-s001.zip › molecules-1173889-supplementary.pdf]

Supplementary Materials

Antioxidant and Antidiabetic Activity of Proanthocyanidins From *Fagopyrum Dibotrys*

Xin Li [1], Jingling Liu [1], Qinxiang Chang [2], Ziyun Zhou [1], Ruilian Han [3] and Zongsuo Liang [1,3,*]

[1] College of Life Sciences, Northwest A & F University, Yangling 712100, China
[2] Institute of Landscape, Taiyuan University, Taiyuan 030032, China
[3] Zhejiang Provincial Key Laboratory of Plant Secondary Metabolism Regulation, College of Life Science and Medicine, Zhejiang Sci-Tech University, Hangzhou 310018, China

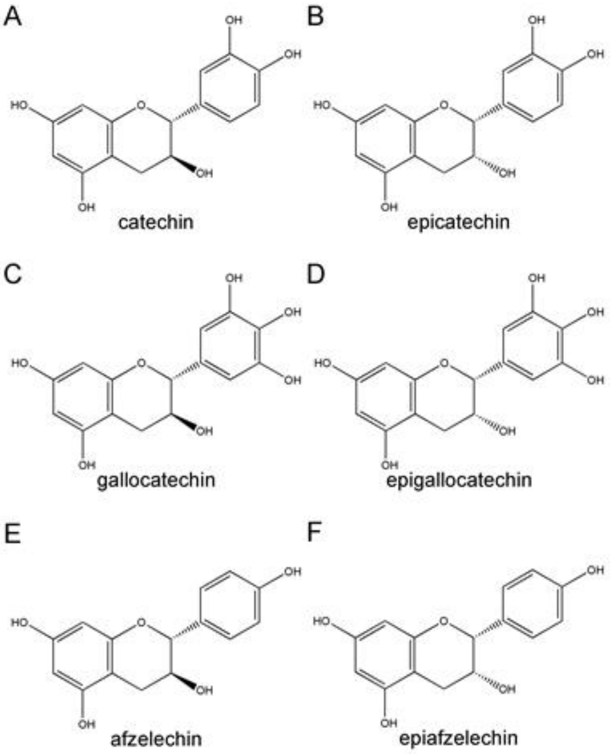

Figure S1. The Chemical structure of typical flavane-3-ol monomers. (**A**), catechin, (**B**), epicatechin, (**C**), gallocatechin, (**D**), epigallocatechin, (**E**), afzelechin, (**F**), epiafzelechin.

Table S1. The abbreviations in this article.

Abbreviation	Full name
^{13}C NMR	^{13}C nuclear magnetic resonance spectroscopy
α-glu	S cerevisiae α-glucosidase
ABTS	2,2-diazo-bis(3-ethyl-benzothiazole-6-sulfonic acid) diammonium salt
AF	Afzelechin
C	Catechin
CG	Catechin gallate
CUPRAC	Cupric ion reducing power
Da	Dalton
DHB	2′5-dihydroxybenzoic acid
DMSO	Dimethyl sulfoxide
DMSO-d_6	Deuterium dimethyl sulfoxide
DPPH	1,1-diphenyl-2-picrylhydrazyl
DW	Dry weight
EAF	Epiafzelechin
EC	Epicatechin
EC_{50}	Half scavenging concentrations
ECG	Epicatechin gallate
EGC	Epigallocatechin
FRAP	Ferric ion reducing antioxidant power
FT-IR	Fourier transform infrared spectroscopy
Gal-G_2-α-CNP	2-choro-4-nitrophenyl-α-galactosyl-maltoside

GC	Gallocatechin
GsPs	Grape seed proanthocyanidins reference standard
HSA	Human salivary α-amylase
IC_{50}	Half inhibitory concentrations
MALDI-TOF MS	Matrix-assisted laser desorption/ionization-time of flight mass spectrometry
mDP	Mean degree of polymerization
NHCM	Neocuproine hydrochloride monohydrate
p-NPG	4-nitrophenyl-α-D-glucopyranoside
PPA	Porcine pancreatic α-amylase
RP-HPLC-ESI-MS	Reversed-phase high-performance liquid chromatog-raphy-electrospray mass spectrometry
TFA	Trifluoroacetic acid
TOPCs	Total proanthocyanidins content
TPTZ	2,4,6-tripyridinyl-1,3,5-triazine
Trolox	6-hydroxy-2,5,7,8-tetramethylchromo-2-carboxylic acid
UV-Vis	Ultraviolet visible spectroscopy

A brief introduction of the seven Polygonaceae plants used in this article.

Fagopyrum dibotrys, also known as golden buckwheat, is a perennial erect herb of genus *Fagopyrum* in Polygonaceae, which mainly grows in the north temperate zone and is widely distributed in China, Kazakhstan, Russia, Ukraine and other regions [10]. *F. dibotrys* has rich nutritional value and health care function, and the rhizome of *F. dibotrys* has a long history of being used as an anti-cancer and anti-inflammatory herb in China [10,13-14].

Fallopia multiflora, also known as "Heshouwu" in Chinese, is a perennial liana of genus *Fallopia* in Polygonaceae, which is widely distributed in southern China, Japan and other regions [45]. *F. multiflora* has been used as a folk medicine in China, korean, Japan, Europe, America, and Australia for the treatment of alopecia and preventing premature graying of the hair for decades. In tradidtion Chinese medicine, *F. multiflora* have been prescribed for detoxification, moistening the intestines, elimination of carbuncles and used an antimalarial agent and laxative. Modern pharmacological studies showed that anti-inflammatory, antitumour, anti-atherosclerosis, and hepatoprotective effects were the main bioactivities of *F. multiflora* extracts. Many compounds including anthraquinones, stilbenes phenolic acids phospholipids, flavonoids and dianthrone derivatives have been isolated from *F. multiflora* with a wide range of bioactivities [45–46].

Polygonum aviculare, also known as "Bianxu" in Chinese, is an annual herb of genus *Polygonum* in Polygonaceae, which is mainly grows in the north temperate zone and is widely distributed throughou China and other asian countries. *Polygonum aviculare* has been used as traditional cuisines and folk medicine for the treatment of stomach pains and diarrhea in various cultures. In traditional Chinese medicine, *P. aviculare* has a long history of been employed to treat cancer, modern studies showed that *P. aviculare* could sensitize chemoresistant cancer cells and enhance the efficacy of some cytostatics, besieds, the extracts of *P. aviculare* are rich in flavonoids, sesquiterpenoids, and tannins and showed potent α-glucosidase inhibitory activity [47–49].

Polygonum orientale, also known as "Hongliao" in Chinese, is an annual herb of genus *Polygonum* in Polygonaceae, which is widely distributed throughout China, Korea, Japan, Russia, the Philippines, India, Europe and Oceania. *P. orientale* have therapeutic effects for dispelling rheumatism, promoting digestion, aiding diuresis and activating blood circulation and widely used in many ethnic groups in China. Modern studies showed that *P. orientale* have a wide range of pharmacological effects, such as anti-oxidative, anti-aging, anti-inflammation, analgesia, anti-myocardial ischemia, anti-abortion, anti-rheumatoid arthritis etc. flavonoids, carboxylic acids, phenolic acids,

amino acids, hydrocarbons, chromones, lignans, volatile oils, amides and other components have been isolated from *P. orientale* [50–51].

Reynoutria japonica, also known as "Huzhang" in Chinese, is a perennial liana of genus *Reynoutria* in Polygonaceae, which is mainly distributed in China, Korea, Japan and other regions. *R. japonica* is used as a functional food in Japan and Korea and is also a well-known traditional antidiabetic herb used in China, both *F. dibotrys* and *R. japonica* are used to treat diabetes in as folk medicine in China, so *F. dibotrys* and *R. japonica* together with other five Polygonaceae plants related to treating diabetes were compared in this study [11].

Rheum officinale, also known as "Yaoyongdahuang" in Chinese, is a perennial liana of genus *Rheum* in Polygonaceae, which is mainly distributed in Shaanxi, Sichuan, Hubei, Guizhou, Yunnan, Henan and other places in China. *R. officinale* is an herbal medicine for the treatment of catharsis, fever, infection, inflammation, diabetes and cancer in China, Japan and Korea. Emodin, chrysophenol, rhein, aloe-emodin physcion etc. were isolated from *R. officinale* and the major pharmacologic constituents are considered to be anthraquinone and bianthrone derivatives, *F. dibotrys*, *R. japonica* and *R. officinale* have been proved to have anti-diabetic activity but their anti-diabetic activity has never been compared together [12].

Rumex acetosa, also known as sorrel in Europe and "Suanmo" in Chinese, is a perennial liana of genus *Rumex* in Polygonaceae, which is mainly distributed throughout China, Korea, Japan, Kazakhstan, Russia, Europe, America and other regions. *R. acetosa* are used as fresh or cooked vegetables in many cultures around the world, and it has been used as folk remedies for gastrointestinal disorders and cutaneous diseases in East Asia. Moderm study showed that *R. acetosa* have potent antioxidant, anti-inflammatory, antiviral, antiproliferative and anti-diabetic activity due to the rich vitamin C, flavonoids, phenolic acids, and proanthocyanidins in *R. acetosa* [52–55].
